# Expression of two non-mutated genetic elements is sufficient to stimulate oncogenic transformation of human mammary epithelial cells

**DOI:** 10.1038/s41419-018-1177-6

**Published:** 2018-11-19

**Authors:** Vijay Pandey, Min Zhang, Mingliang You, Weijie Zhang, Rumei Chen, Wei Zhang, Lan Ma, Zheng-Sheng Wu, Tao Zhu, Xiu Qin Xu, Peter E. Lobie

**Affiliations:** 1 Tsinghua-Berkeley Shenzhen Institute, Tsinghua University, Shenzhen, Guangdong PR China; 2 Hefei National Laboratory for Physical Sciences at Microscale and School of Life Sciences, University of Science and Technology of China, Hefei, Anhui PR China; 3 Cancer Science Institute of Singapore, National University of Singapore, Singapore, Singapore; 4 Department of Breast Surgery, The First Affiliated Hospital of Jinan University, Jinan University, Tianhe District, Guangzhou, Guangdong PR China; 5 Guangdong Provincial Key Laboratory of Malignant Tumor Epigenetics and Gene Regulation, Breast Tumor Center, Sun Yat-Sen Memorial Hospital, Sun Yat-Sen University, Guangzhou, PR China; 6 Department of Pathology, Anhui Medical University, Hefei, Anhui PR China; 7 Institute of Stem Cell and Regenerative Medicine, Medical College, Xiamen University, Fujian, PR China

## Abstract

Trefoil factor 3 (TFF3) expression is positively associated with advanced clinicopathological features of mammary carcinoma (MC). Herein, we provide evidence for a functional role of TFF3 in oncogenic transformation of *immortalized*, but otherwise normal human mammary epithelial cells (HMECs), namely, HMEC-*hTERT*, MCF10A, and MCF12A. Forced expression of TFF3 in *immortalized*-HMECs enhanced cell proliferation, cell survival, anchorage-independent growth, produced highly disorganised three-dimensional (3D) acinar structures and generated tumours in immunocompromised mice. Forced expression of TFF3 in *immortalized*-HMECs stimulated STAT3 activity that was required for TFF3-stimulated cell proliferation, survival, and anchorage-independent growth. TFF3 specifically utilised STAT3 activity to govern a transcriptional program, which was required for TFF3-stimulated oncogenic transformation of *immortalized*-HMECs, including transcriptional upregulation of *CCND1* and *BCL2*. *siRNA*-mediated depletion or functional inhibition of STAT3 significantly inhibited the TFF3-stimulated transcription of *CCND1* and *BCL2* and oncogenicity in *immortalized*-HMECs. Furthermore, DOX-inducible expression of TFF3 in HMEC-*hTERT* cells also permitted anchorage-independent growth and produced disorganized acinar structures in 3D Matrigel culture. Removal of DOX-induced expression of TFF3 in HMEC-*hTERT* cells, previously grown with DOX, resulted in efficient normalisation of the disorganized acinar architecture and attenuated cell viability in Matrigel culture. Cumulatively, these findings suggest that TFF3 is a potent oncogene and its increased expression along with *hTERT* in HMECs is sufficient to produce oncogenic transformation.

## Introduction

A combination of cell proliferation and cell survival have been postulated to provide a platform for the oncogenic transformation of normal cells^1,2^. It was previously demonstrated that oncogenic transformation of human mammary epithelial cells (HMECs) requires the expression of at least three genetic elements, including *hTERT* (the catalytic subunit of human telomerase), the *SV40* large T antigen, and an oncogenic form of the *HRAS* gene^3^. However, delineating more physiologically and aetiologically relevant genes involved in oncogenic transformation of mammary epithelial cells will provide a more significant understanding of this disease process.

Human trefoil factor 3 (TFF3) is a protein belonging to the trefoil factor family (TFF) of proteins and it shares homology with 2 other members namely, TFF1 and TFF2^4^. TFF3 expression is predominantly observed in the epithelium of the gastrointestinal tract, where it promotes repair of the mucosa after injury^5^. TFF3 has emerged as a validated and functionally potent target in female reproductive-related malignancies^6–9^. Low/absent expression of TFF3 is observed in ductal epithelial cells of the normal mammary gland. However, significantly increased expression has been observed in both in situ and invasive mammary carcinomas (MC)^6–8^. Clinicopathological analyses demonstrated that TFF3 expression is positively correlated with advanced features of disease, such as tumour size, microvessel density, higher disease grade and metastases^8,10^. Expression of TFF3 is also highly significantly associated with poor prognosis in MC patients^8^. In one MC patient cohort, TFF3 expression was observed in 44% of ER-negative MC suggestive that TFF3 may also function in this recalcitrant subtype of MC^8^. TFF3 has been suggested to be a promiscuous ligand that activates a multitude of signalling pathways, including CXCR4/7, HER1-4, MET, SRC, and IGFR1; and also promotes down-stream activity of MAPK, NF-κB, PI3K-AKT, and STAT3^8,11–18^ with resultant cell survival, cell proliferation, angiogenesis, and metastatic dissemination^7–9^. However, the role of TFF3 in the oncogenic transformation process is not defined.

Herein, we have demonstrated the capacity of TFF3 to stimulate oncogenic transformation in three different *immortalized*-HMEC lines and delineated the mechanisms involved.

## Results

### Forced expression of TFF3 in *immortalized*-HMECs stimulates an oncogenic phenotype and tumour formation

**Fig. 1:**
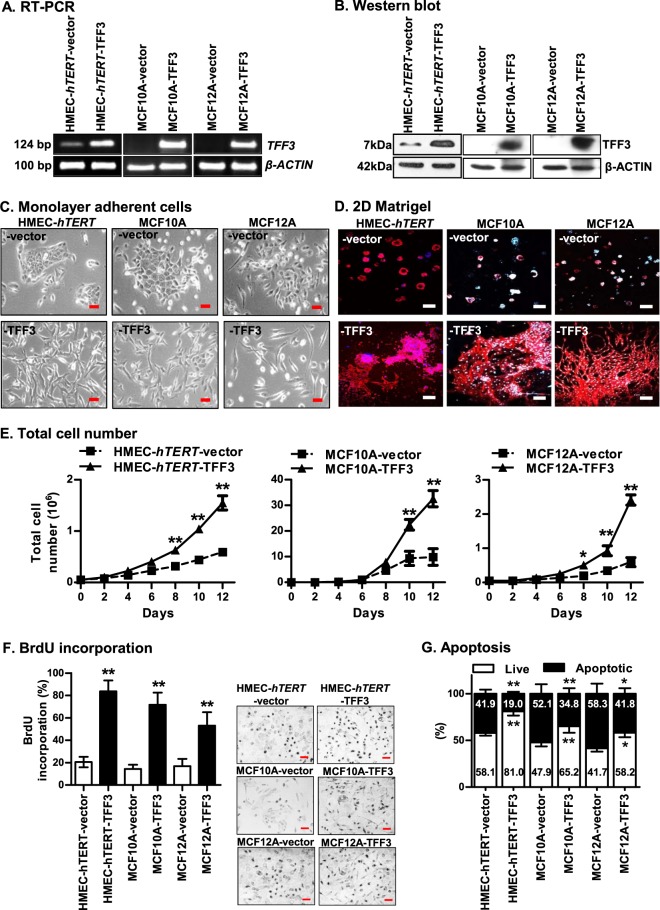
Forced expression of TFF3 in *immortalized*-HMECs stimulates phenotypic changes and cell proliferation. HMEC-*hTERT*, MCF10A and MCF12A cells were transfected with the *pIRESneo3-TFF3* expression construct to generate the corresponding stable cell lines with forced expression of TFF3; a *pIRESneo3* construct was used as vector control as described in Materials and methods^7,8^. Stable clones were designated as HMEC-*hTERT*-vector and HMEC-*hTERT*-TFF3; MCF10A-vector and MCF10A-TFF3; and MCF12A-vector and MCF12A-TFF3. The levels of TFF3 expression in *immortalized*-HMECs with forced expression of TFF3 and their vector control cells was demonstrated using **a** RT-PCR and **b** western blot. Soluble whole-cell extracts were run on an SDS-PAGE and immunoblotted using rabbit polyclonal anti-TFF3 antibody as described in Materials and methods. β-actin was used as input control. The sizes of *PCR* product in base pair (bp) are shown on the left side and detected protein bands size in kDa are shown on the right side. Among *immortalized*-HMECs, HMEC-*hTERT* cells exhibit-deficient endogenous levels of TFF3 *mRNA* and protein, whereas, endogenous expression of TFF3 was not detected in MCF10A and MCF12A cells by RT-PCR and western blot. **c** Representative phase-contrast microscopic images of *immortalized*-HMECs with forced expression of TFF3 and their vector control cells on monolayer culture. Images were captured under ×200 magnification. **d** Representative confocal laser scanning microscopy images of *immortalized*-HMECs with forced expression of TFF3 and their vector control cells cultured on Matrigel (2D). Images were captured under ×100 magnification. **e** Total cell number, **f** BrdU incorporation, and **g** apoptotic cell death of *immortalized*-HMECs with forced expression of TFF3 and their vector control cells. Forced expression of TFF3 in *immortalized*-HMECs significantly abrogated apoptotic cell death as a consequence of serum deprivation when compared to their vector control cells. All assays were performed as described in Material and Methods. Column or chart point is mean of triplicate experiments; bars, ±SD. **P < 0.001, *P < 0.05

To determine the oncogenic transforming capacity of TFF3, we utilized *hTERT*-*immortalized* HMEC (HMEC-*hTERT*^3^) and two spontaneously *immortalized*-HMECs (MCF10A and MCF12A). Forced expression of TFF3 in *immortalized*-HMECs was demonstrated at both the *mRNA* and protein levels (Fig. 1a, b). HMEC-*hTERT*-TFF3 cells exhibited an elongated morphology, with loss-of-cell–cell contact, and formed multiple cellular protrusions (Fig. 1c). In contrast, HMEC-*hTERT*-vector cells exhibited epithelial characteristics and grew as a defined group of colonies with extensive cell-to-cell contact. In prolonged culture on Matrigel (2D), HMEC-*hTERT*-TFF3 cells grew in a stellate organization with cords of cells extending from the colonies (Fig. 1d). In contrast, HMEC-*hTERT*-vector cells formed tightly grouped colonies when cultured on Matrigel. HMEC-*hTERT*-TFF3 cells exhibited increased total cell number (TCN) compared to HMEC-*hTERT*-vector cells in monolayer culture (Fig. 1e). HMEC-*hTERT*-TFF3 cells exhibited both increased entry to S-phase and abrogated apoptotic cell death as a consequence of serum deprivation when compared to HMEC-*hTERT*-vector cells (Fig. 1f, g). Similarly, the TFF3-stimulated phenotypic changes, increased TCN, increased mitogenesis and decreased apoptosis were also observed in MCF10A or MCF12A cells (Fig. 1).

**Fig. 2:**
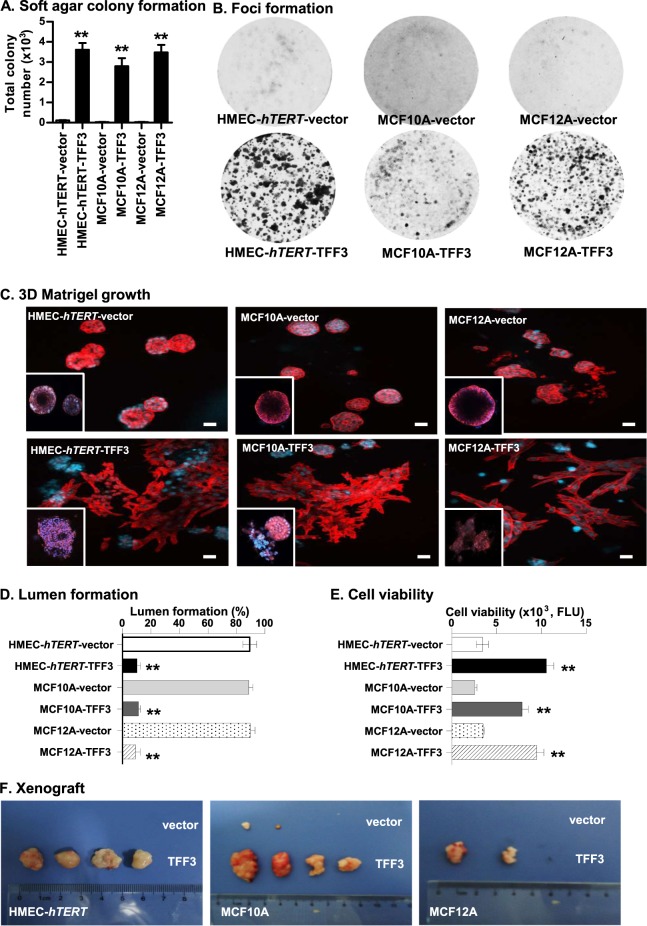
Forced expression of TFF3 in *immortalized*-HMECs stimulates anchorage-independent growth, growth in ex vivo Matrigel (3D) culture, and tumour formation in xenografts. **a** Soft agar colony formation by a single *immortalized*-HMECs with forced expression of TFF3 and their vector control cells. A total number of colonies was calculated after cells cultured in the complete medium over a period of 18 days. **b** Foci formation by *immortalized*-HMECs with forced expression of TFF3 and their vector control cells after cultured in the complete medium over a period of 18 days. **c** Confocal laser scanning microscopy visualization of colonies generated by a single *immortalized*-HMECs with forced expression of TFF3 and their vector control cells after cultured in three-dimensional (3D) Matrigel over a period of 18 days. Forced expression of TFF3 results in filling of the luminal space and loss-of-proliferative arrest in the human mammary acinus (focused image in down left-corner of representative images). Confocal microscopy was performed using Rhodamine-conjugated phalloidin to visualize F-actin filaments (red colour), and nuclei were counterstained with DAPI (blue colour). Images were captured under ×400 magnification. **d** Acinar (lumen) structures generated by a single *immortalized*-HMECs with forced expression of TFF3 and their vector control cells were calculated and tabulated after cultured in 3D Matrigel over a period of 18 days. **e** Cell viability in colonies generated by *immortalized*-HMECs with forced expression of TFF3 and their vector control cells after cultured in 3D Matrigel over a period of 18 days. **f** Representative images of resected tumour mass formed by *immortalized*-HMECs with forced expression of TFF3 and their vector control cells in xenografts. All assays were performed as described in Material and Methods. The column is mean of triplicate experiments; bars, ±SD. **P < 0.001, *P < 0.05

An acquired capacity for anchorage-independent growth and the formation of disorganized three-dimensional (3D) acinar structures with a filled lumen is a hallmark of glandular epithelial tumours^19–22^. HMEC-*hTERT*-TFF3 cells exhibited an enhanced capacity for soft agar colony formation compared to HMEC-*hTERT*-vector cells (Fig. 2a; SI 1A). Moreover, HMEC-*hTERT*-TFF3 cells exhibited increased TCN in suspension culture (SI 1B) and enhanced foci formation (Fig. 2b) compared to HMEC-*hTERT*-vector cells. HMEC-*hTERT*-TFF3 cells also formed disorganized multi-acinar undifferentiated structures without lumina (Fig. 2c). More than 80% of HMEC-*hTERT*-TFF3 cells exhibited such large irregular multi-acinar structures with a filled lumen (Fig. 2d). In contrast, HMEC-*hTERT*-vector cells formed growth-arrested spheroidal acini. The acinar units consisted of a layer of cells attached to the matrix and surrounding a hollow lumen (Fig. 2c). HMEC-*hTERT*-TFF3 cells also exhibited increased 3D cell viability compared to their vector control cells (Fig. 2e). Similarly, TFF3-stimulated anchorage-independent growth and phenotypic changes in Matrigel culture were also observed in MCF10A and MCF12A cells (Fig. 2). Neutralization of TFF3 by anti-TFF3 polyclonal antibody completely abrogated the effect of forced expression of TFF3 in *immortalized*-HMECs grown in 3D Matrigel (SI 1C).

The expression of proto-oncogenes in *immortalized*-human epithelial cells, such as CCND1 or FRA-1, is sufficient to stimulate the anchorage-independent growth, yet are insufficient to result in tumour formation in mice^23,24^. We, therefore, implanted both vector control and *immortalized*-HMECs with forced expression of TFF3 into the first mammary (axillary) fat pad of intact athymic female mice with use of Matrigel as a vehicle. HMEC-*hTERT*-TFF3 cells injected with Matrigel as vehicle formed large palpable tumours (average size, 804.51 ± 130.71 mm^3^) in 4 of 8 of the animals injected, whereas HMEC-*hTERT*-vector cells did not form tumours (0/8) (Fig. 2f). The latency of macroscopic tumour appearance was ~4 weeks. Similarly, injection of MCF12A-TFF3 cells with Matrigel as vehicle formed large palpable tumours at 6-week (average size, 616.02 ± 113.94 mm^3^) in 2 of 8 animals, whereas MCF12A-vector cells did not form tumours (0/8). Injection of MCF10A-TFF3 cells with Matrigel as the vehicle also formed large palpable tumours (average size, 682.51 ± 183.05 mm^3^) in 4/8 injected animals. Two animals (2/8) injected with MCF10A-vector cells also formed small size tumours (average size, 32.51 ± 19.79 mm^3^), most likely a consequence of the spontaneous transformation of this cell line as described previously^25^. Thus, forced expression of TFF3 in *immortalized*-HMECs stimulates an oncogenic phenotype with tumour formation in vivo.

### TFF3 mediates its oncogenic activities in *immortalized*-HMECs through STAT3 activity

**Fig. 3:**
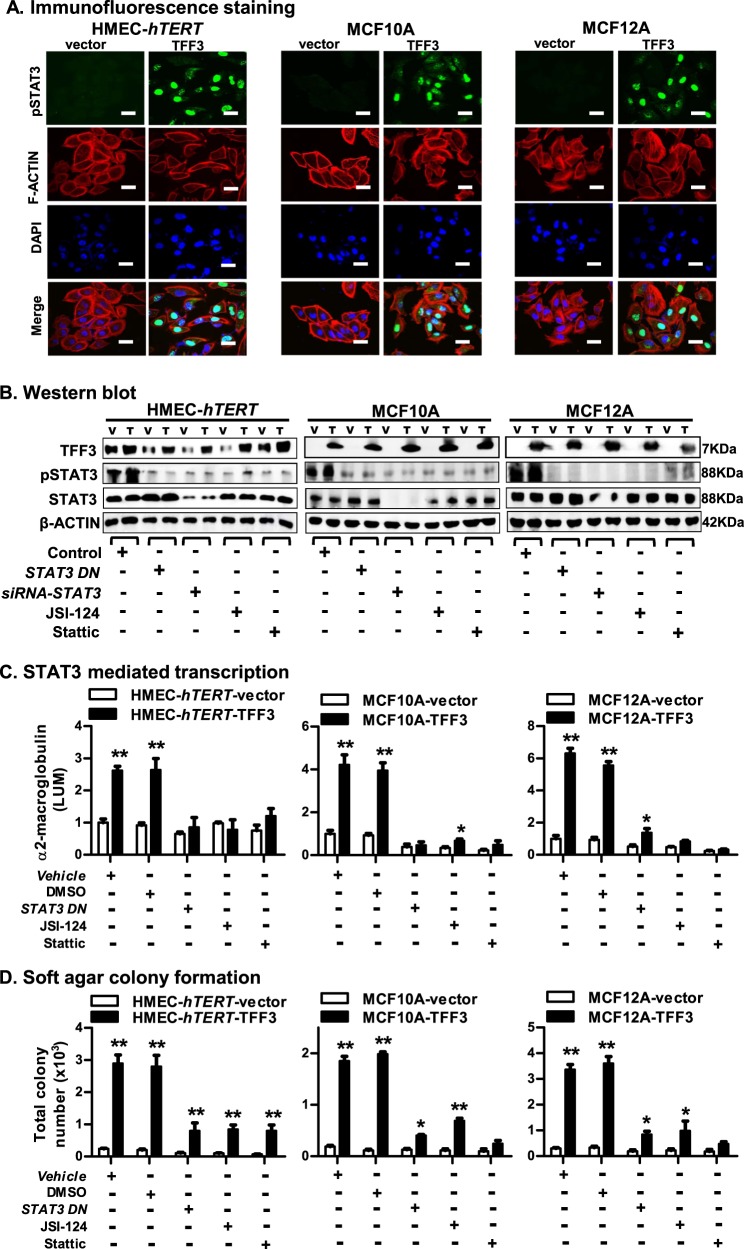
TFF3 mediates its oncogenic activities in *immortalized*-HMECs through STAT3 activity. **a** Confocal microscopic scanning of pSTAT3 and F-actin arrangement in *immortalized*-HMECs with forced expression of TFF3 and their vector control cells. Green colour denoted pSTAT3, red colour denoted F-actin, and blue colour denoted nuclei stained with DAPI. Representative images were captured under oil immersion ×600 magnification. **b** Western blot analysis was used to assess the protein levels of TFF3, pSTAT3 (at Tyr 705), and STAT3 in *immortalized*-HMECs with forced expression of TFF3 and their vector control cells, after inhibition of STAT3. Inhibition of STAT3 executed using transient-transfection of *siRNA-STAT3* or *STAT3-*dominant negative (DN); or on exposure to JSI-124 (0.2 µM) or Stattic (2 µM) inhibitor. Densitometric analysis demonstrated that the levels of pSTAT3 (Tyr 705) increased 39 ± 4% in HMEC-*hTERT*-TFF3, 25 ± 6% in MCF10A-TFF3, and 20 ± 3% in MCF12A-TFF3 cells compared to their vector control cells. Soluble whole cellular extracts were run on an SDS-PAGE and immunoblotted as described in materials and methods. β-actin was used as an input control for cell lysate. The sizes of detected protein bands in kDa are shown on the right side. **c** STAT3-mediated transcription was examined using luciferase activity of *α2-M* promoter activity in *immortalized*-HMECs with forced expression of TFF3 and their vector control cells after transient-transfection of *STAT3 DN* or on exposure to JSI-124 (0.2 µM) or Stattic (2 µM) inhibitor. **d** Soft agar colony formation by *immortalized*-HMECs with forced expression of TFF3 and their vector control cells after transient-transfection of *STAT3 DN* or on exposure to JSI-124 (0.2 µM) or Stattic (2 µM) inhibitor. The luciferase assay and soft agar colony formation assay was performed as described in material and methods. The column is mean of triplicate experiments; bars, ±SD. **P < 0.001, *P < 0.05

TFF3 has previously been demonstrated to promote oncogenicity and metastatic seeding of MC cells through enhanced STAT3 activity^7,8^. We therefore examined STAT3 activity in *immortalized*-HMECs with forced expression of TFF3. Predominantly nuclear immunoreactivity for pSTAT3 (Y705) was observed in HMEC-*hTERT*-TFF3 cells (Fig. 3a). In contrast, HMEC-*hTERT*-vector cells displayed very low or negative-pSTAT3 staining in the cytoplasm of cells, whereas no pSTAT3 was observed in the nuclei of the cells. Using western blot analysis, we also confirmed that HMEC-*hTERT*-TFF3 cells exhibited markedly increased pSTAT3 activity compared to HMEC-*hTERT*-vector cells. The level of total STAT3 did not differ in HMEC-*hTERT* cells with either forced expression of TFF3 or their vector control. TFF3 stimulation of pSTAT3 levels was also observed in MCF10A or MCF12A cells (Fig. 3a).

We further examined the levels of pSTAT3 in HMEC-*hTERT*-TFF3 and HMEC-*hTERT*-vector cells after inhibition of STAT3 either by transfection of small interfering (si) *RNA* targeting *STAT3 (siRNA-STAT3) or a STAT3* dominant-negative mutant (*STAT3-DN*); or on exposure to pharmacological STAT3 inhibitors JSI-124 or Stattic. Both basal and the TFF3-stimulated pSTAT3 level were decreased in HMEC-*hTERT* cells after depletion or inhibition of STAT3 (Fig. 3b). HMEC-*hTERT*-TFF3 cells also exhibited increased luciferase activity of *α2-macroglubulin* (*α2-M*, a STAT3-mediated transcriptional activation) promoter compared to HMEC-*hTERT*-vector cells (Fig. 3c). TFF3-stimulated *α2-M* promoter activity in HMEC-*hTERT* cells was also prevented by the depletion or inhibition of STAT3. Similarly, the forced expression of TFF3 in MCF10A or MCF12A cells also exhibited augmented pSTAT3 levels and *α2-M* promoter activity, whereas depletion or inhibition of STAT3 attenuated the TFF3-stimulated STAT3 activity and STAT3-mediated transcriptional activation (Fig. 3b, c).

**Fig. 4:**
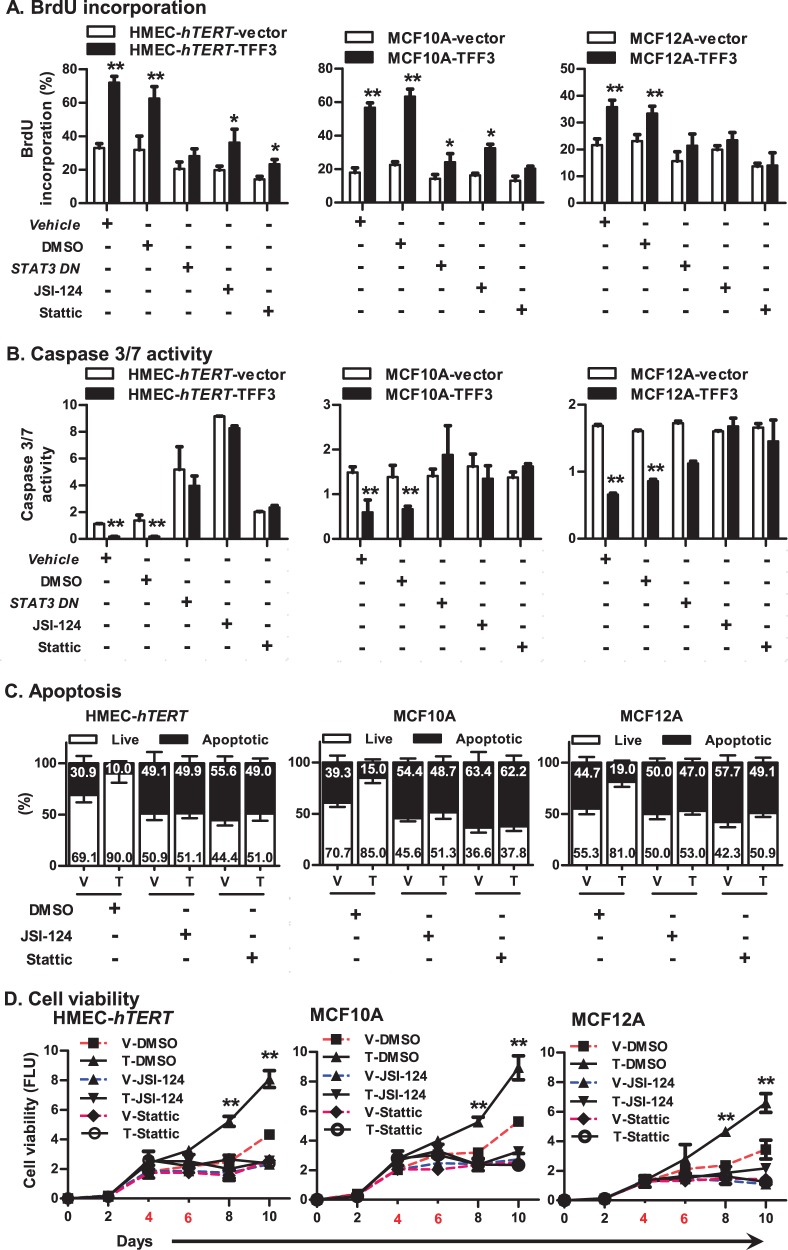
TFF3 utilizes STAT3 activity to stimulate mitogenesis, promote resistance against apoptosis, and increase cell viability of *immortalized*-HMECs in 3D Matrigel culture. **a** BrdU incorporation in *immortalized*-HMECs with forced expression of TFF3 and their vector control cells after transient-transfection of *STAT3 DN* or on exposure to JSI-124 (0.2 µM) or Stattic (2 µM) inhibitor. **b** Caspase 3/7 activity in *immortalized*-HMECs with forced expression of TFF3 and their vector control cells after transient-transfection of *STAT3 DN* or on exposure to JSI-124 (0.2 µM) or Stattic (2 µM) inhibitor. **c** Apoptotic cell death in *immortalized*-HMECs with forced expression of TFF3 and their vector control cells upon on exposure to JSI-124 (0.2 µM) or Stattic (2 µM) inhibitor. **d** Cell viability of *immortalized*-HMECs with forced expression of TFF3 and their vector control cells in 3D Matrigel culture monitored by AlamarBlue® assay. Inhibition of STAT3 was executed upon exposure to JSI-124 (0.2 µM) or Stattic (2 µM) inhibitor. Control cells were exposed to DMSO. Numbers denoted with red colour in *X*-axis indicate the day of inhibitor exposure. All assays were performed as described in Material and Methods. Column or chart point is mean of triplicate experiments; bars, ±SD. **P < 0.001, *P < 0.05

We next examined the functional consequences of STAT3 inhibition in HMEC-*hTERT*-TFF3 and HMEC-*hTERT*-vector cells, either by transfection of *STAT3 DN* or on exposure to *JSI*-124 or Stattic. The TFF3-stimulated capacity for colonization in soft agar of HMEC-*hTERT* cells was considerably reduced after inhibition of STAT3 (Fig. 3d). Also, the TFF3-stimulated entry to S-phase in HMEC-*hTERT* cells was considerably abrogated after inhibition of STAT3 (Fig. 4a). Concomitantly, TFF3-stimulated repression of caspase 3/7 activity was also prevented after inhibition of STAT3 in HMEC-*hTERT* cells. However, both HMEC-*hTERT*-vector or HMEC-*hTERT*-TFF3 cells exhibited increased caspase 3/7 activity after inhibition of STAT3 (Fig. 4b). Exposure of HMEC-*hTERT* cells to STAT3 inhibitors also abrogated the TFF3-stimulated cell survival (Fig. 4c). Furthermore, as demonstrated in Fig. 2d, HMEC-*hTERT*-TFF3 cells exhibited increased cell viability compared to HMEC-*hTERT*-vector cells when cultured in 3D-Matrigel over a period of 10 days (Fig. 4d). Upon exposure to JSI-124 or Stattic on the fourth and sixth day, the stimulatory effect of TFF3 on cell viability was prevented in HMEC-*hTERT* cells grown in 3D-Matrigel. Similar directional changes in anchorage-independent growth, S-phase entry (cell cycle), apoptotic cell death and cell viability in 3D-Matrigel was observed in MCF10A or MCF12A cells after inhibition of STAT3 (Fig. 4). As previously described in mammary carcinoma cells^8^, we also herein demonstrated that forced expression of TFF3 in *immortalized*-HMEC cells enhanced phosphorylation of cSRC that subsequently increased STAT3 activity to promote cell viability (SI 2). Thus, TFF3 utilizes STAT3 activity to execute its oncogenic activities in *immortalized*-HMECs.

### STAT3 governs the transcriptional switch in the TFF3-stimulated oncogenic transformation of *immortalized*-HMECs

Using quantitative-PCR analyses, we determined the *mRNA* level of genes associated with cell-cycle progression and cell survival of *immortalized*-HMECs with forced expression of TFF3^3,22^. Forced expression of TFF3 in HMEC-*hTERT* cells increased the *mRNA* level of *CCND1*, *CCNE1*, and *CDC25A*, genes that positively regulate cell-division^26^ (SI 3). The *mRNA* level of *CDKN2A* and *CDKN1A*, an inhibitor of *CDK4* and *CDK2*, respectively^26^, was decreased in HMEC-*hTERT*-TFF3 cells. Moreover, forced expression of TFF3 in HMEC-*hTERT* cells increased the *mRNA* level of genes, *BCL2* and *BCLXL*/*BCL2L1*, which possess pro-survival activities in epithelial cells^27^. Concomitantly, the *mRNA* level of genes encoding *BA*X and *CASP7*, promoting the apoptotic process were decreased in HMEC-*hTERT*-TFF3 cells^27^. Interestingly, the *mRNA level* of *hTERT* was also increased in HMEC-*hTERT*-TFF3 cells indicative of a function of TFF3 in *immortalization* of HMECs^28^. Of note, forced expression of TFF3 in HMEC-*hTERT* cells also exhibited increased *mRNA* level of genes positively associated with angiogenesis^9^ and metastatic processes of MC and as previously reported^8,22^. Similar directional changes in the *mRNA* level of genes involved in cell-cycle progression and cell survival were observed in MCF10A or MCF12A cells with forced expression of TFF3 (SI 3).

**Fig. 5:**
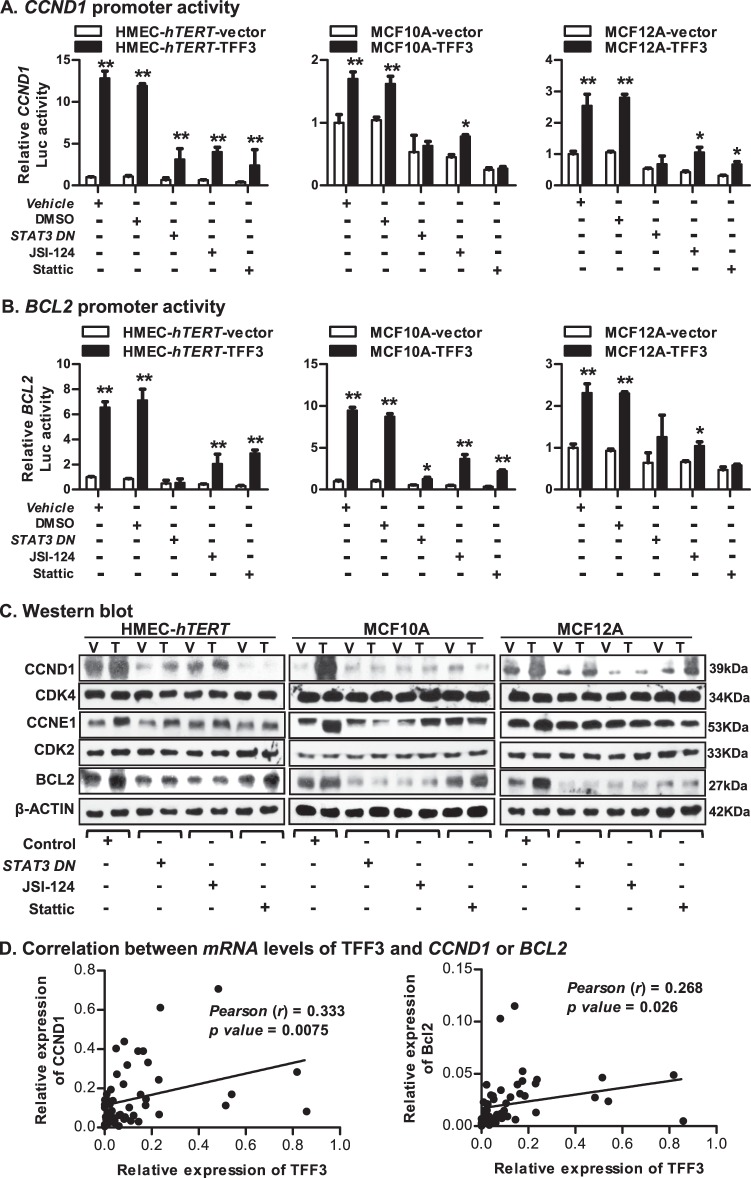
STAT3 governs the transcriptional switch in the TFF3-stimulated oncogenic transformation of *immortalized*-HMECs. **a** Luciferase activity of the *CCND1* promoter in *immortalized*-HMECs with forced expression of TFF3 and their vector control cells after transient-transfection of *STAT3 DN* or on exposure to JSI-124 (0.2 µM) or Stattic (2 µM) inhibitor. **b** Luciferase activity of the *BCL2* promoter in *immortalized*-HMECs with forced expression of TFF3 and their vector control cells after transient-transfection of *STAT3 DN* or on exposure to JSI-124 (0.2 µM) or Stattic (2 µM) inhibitor. **c** Western blot analysis was used to assess the protein levels of CCND1, CDK4, CCNE1, CDK2, and BCL2 in *immortalized*-HMECs with forced expression of TFF3 and their vector control cells, after inhibition of STAT3. Inhibition of STAT3 executed using transient-transfection of *STAT3 DN* or on exposure to JSI-124 (0.2 µM) or Stattic (2 µM) inhibitor. Densitometric analysis demonstrated that HMEC-*hTERT*-TFF3 cells exhibited increased protein levels of CCND1 (49 ± 8%), CCNE1 (95 ± 18%), and BCL2 (114 ± 17%) compared to HMEC-*hTERT*-vector cells. MCF10A-TFF3 cells exhibited increased protein levels of CCND1 (4902 ± 263%), CCNE1 (125 ± 27%), and BCL2 (79 ± 16%) compared to MCF10A-vector cells. MCF12A-TFF3 cells exhibited increased protein levels of CCND1 (92 ± 25%), CCNE1 (47 ± 12%), and BCL2 (137 ± 25%) compared to MCF12A-vector cells. Soluble whole cellular extracts were run on an SDS-PAGE and immunoblotted as described in materials and methods. β-actin was used as an input control for cell lysate. The sizes of detected protein bands in kDa are shown on the right side. **d** Correlation between *mRNA* levels of *TFF3* and *CCND1* or *BCL2* in the MC cohort. Pearson’s *χ*^2^-test was used to compare the differences between groups. Statistical significance was assessed by using an unpaired two-tailed Student’s *t*-test (*P* < 0.05 was considered as significant) using GraphPad Prism 5. The luciferase assay was performed as described in Material and Methods. The column is mean of triplicate experiments; bars, ±SD. **P < 0.001, *P < 0.05

STAT3 governs a transcriptional switch, which pivotally regulates cell proliferation and cell survival^8,29,30^. We therefore assessed the luciferase reporter activity of the *CCND1* or *BCL2* promoters in HMEC-*hTERT*-TFF3 and HMEC-*hTERT*-vector cells after inhibition of STAT3 by transfection of *STAT3-DN* or on exposure to JSI-124 or Stattic (Fig. 5a, b). As expected, HMEC-*hTERT*-TFF3 cells exhibited increased *CCND1* or *BCL2 promoter* activities compared to HMEC-*hTERT*-vector cells. The TFF3-stimulated promoter activities of *CCND1* or *BCL2* were abrogated in HMEC-*hTERT* cells after inhibition of STAT3. HMEC-*hTERT*-TFF3 cells also exhibited increased protein levels of CCND1, CCNE1, and BCL2 compared to HMEC-*hTERT*-vector cells (Fig. 5c). Both basal and TFF3-stimulated protein levels of CCND1, CCNE1, and BCL2 were markedly decreased in HMEC-*hTERT* cells after inhibition of STAT3. In contrast, protein levels of cell cycle regulators, CDK4 (for CCND1) and CDK2 (for CCNE1) in HMEC-*hTERT* cells were not changed either with forced expression of TFF3 or after inhibition of STAT3 (Fig. 5c). Similarly, the TFF3-stimulated promoter activities of *CCND1* or *BCL2* and the protein levels of CCND1, CCNE1, and BCL2 in MCF10A or MCF12A cells were also attenuated after inhibition of STAT3 (Fig. 5a–c). Concordantly, we observed a modest but a significant correlation between the *mRNA* levels of *TFF3* with *CCND1* or *BCL2* in a MC patient cohort^8^ (Fig. 5d).

**Fig. 6:**
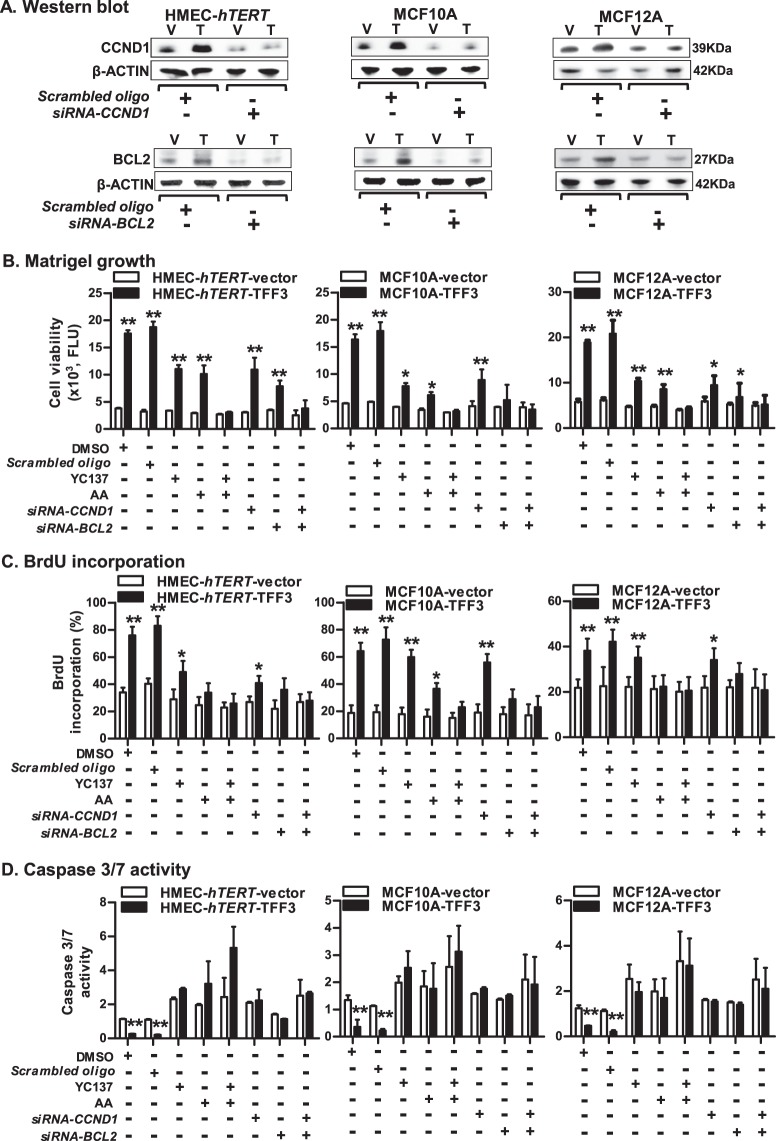
CCND1 and BCL2, both are essential for TFF3-driven oncogenic transformation of *immortalized*-HMECs. **a** Western blot analysis was used to assess the levels of CCND1 and BCL2 in *immortalized*-HMECs with forced expression of TFF3 and their vector control cells after *siRNA-*mediated depletion of CCND1 or BCL2. A control cell was transiently transfected with scrambled oligo. Soluble whole cellular extracts were run on an SDS-PAGE and immunoblotted as described in materials and methods. β-actin was used as an input control for cell lysate. The sizes of detected protein bands in kDa are shown on the right side. **b** Cell viability of *immortalized*-HMECs with forced expression of TFF3 and their vector control cells in 3D Matrigel culture after depletion/inhibition of CCND1 and BCL2. Depletion of CCND1/BCL2 was executed using *CCND1-siRNA*/ *BCL2-siRNA*, respectively. Inhibition of CCND1 or BCL2 was executed upon exposure to Arcyriaflavin A (AA) (50 nM) or YC137 (1 µM) inhibitor, respectively. **c** BrdU incorporation in *immortalized*-HMECs with forced expression of TFF3 and their vector control after depletion/inhibition of CCND1 and BCL2. Depletion of CCND1/BCL2 was executed using *CCND1-siRNA*/ *BCL2-siRNA*, respectively. Inhibition of CCND1 or BCL2 was executed upon exposure to Arcyriaflavin A (AA) (50 nM) or YC137 (1 µM) inhibitor, respectively. **d** Caspase 3/7 activity in *immortalized*-HMECs with forced expression of TFF3 and their vector control cells after depletion/inhibition of CCND1 and BCL2. Depletion of CCND1/BCL2 was executed using *CCND1-siRNA*/ *BCL2-siRNA*, respectively. Inhibition of CCND1 or BCL2 was executed upon exposure to Arcyriaflavin A (AA) (50 nM) or YC137 (1 µM) inhibitor, respectively. All assays were performed as described in Material and Methods. The column is mean of triplicate experiments; bars, ±SD. **P < 0.001, *P < 0.05

To assess the necessity of CCND1 or BCL2 protein in TFF3-stimulated oncogenic transformation of *immortalized*-HMECs, we next utilized *siRNA*-mediated depletion of *CCND1* and *BCL2* (Fig. 6a); or Arcyriaflavin A (AA) (specific to CCND1/CDK4) or YC137 (specific to BCL2). The TFF3-stimulated cell viability of HMEC-*hTERT* cells in Matrigel was significantly decreased after depletion or inhibition of either CCND1 or BCL2 (Fig. 6b). However, the TFF3-stimulated cell viability of HMEC-*hTERT* cells was abrogated after combined depletion or inhibition of CCND1 and BCL2. Similar directional changes were observed in cell viability of MCF10A or MCF12A cells in Matrigel culture after combined inhibition of CCND1 and BCL2 (Fig. 6b). We also examined the effect of depletion or inhibition of CCND1 and BCL2 on cell cycle S-phase entry and caspase 3/7 activity in *immortalized*-HMECs with forced expression of TFF3 (Fig. 6c, d). TFF3-stimulated S-phase entry in HMEC-*hTERT* cells was considerably decreased after depletion or inhibition of either CCND1 or BCL2. Moreover, TFF3-stimulated S-phase entry in HMEC-*hTERT* cells was abolished after combined depletion or inhibition of CCND1 and BCL2 (Fig. 6c). Concomitantly, the TFF3-dependent repression of caspase 3/7 activity in HMEC-*hTERT* cells was prevented, and both HMEC-*hTERT*-TFF3 or HMEC-*hTERT*-vector cells exhibited increased caspase 3/7 activity after depletion or inhibition of CCND1 and BCL2 (Fig. 6d). Similar directional changes were observed in S-phase entry and caspase 3/7 activities in MCF10A and MCF12A cells after combined depletion or inhibition of CCND1 and BCL2 (Fig. 6c, d). Thus, STAT3 governs transcriptional activities of *CCND1* and *BCL2*, which are essential for TFF3-stimulated oncogenic transformation of *immortalized*-HMECs.

### TFF3-stimulated oncogenic transformation is reversible

**Fig. 7:**
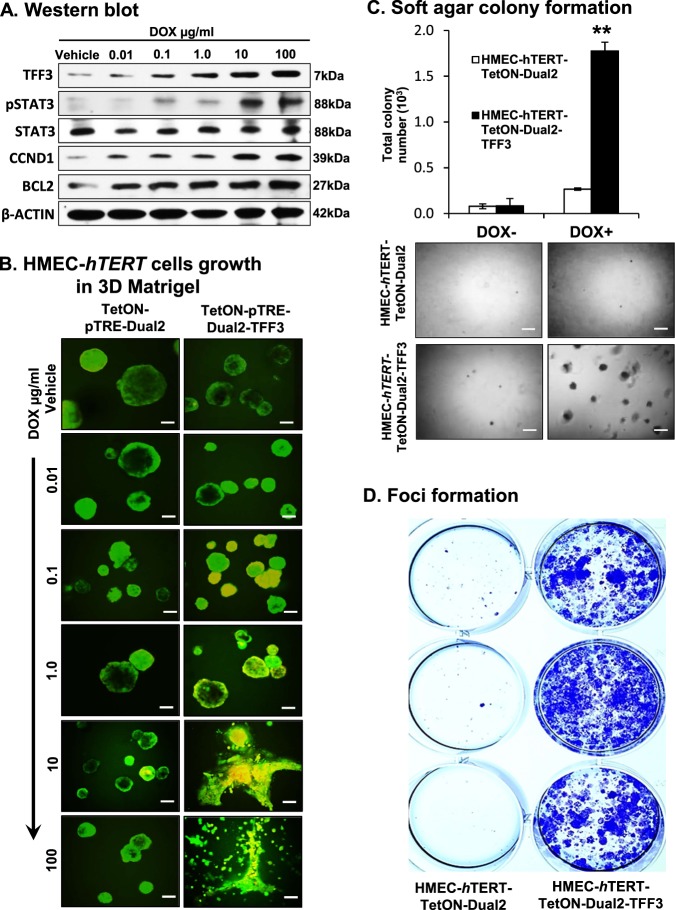
Inducible-TFF3 expression in HMEC-*hTERT* cells increased TFF3 expression and stimulated oncogenic phenotype in 3D Matrigel and capacity for anchorage-independent growth. **a** Western blot analysis was performed to assess the levels of TFF3, pSTAT3, CCND1, BCL2, and STAT3 in HMEC-*hTERT*-TetON-Dual2-TFF3 cells after exposure to increasing concentration of DOX (0.01–100 µg/ml). Soluble whole cellular extracts were run on an SDS-PAGE and immunoblotted as described in Materials and methods. β-actin was used as an input control for cell lysate. The sizes of detected protein bands in kDa are shown on the right side. **b** Confocal laser scanning microscopic cross-sections of the mammary acinar structured formed by HMEC-*hTERT*-TetON-Dual2-TFF3 and HMEC-*hTERT*-TetON-Dual2 cells cultured three-dimensional Matrigel on exposure to increasing concentration of DOX 0.01–100 µg/ml. Green colour denotes ZsGreen1, and red colour denotes mCherry. Consistent with western blot results obtained herein, elevated levels of TFF3 expression was observed after exposure to DOX. Capacity for anchorage-independent of HMEC-*hTERT*-TetON-Dual2-TFF3 cells and HMEC-*hTERT*-TetON-Dual2 cultured in complete medium with or without DOX (1 µg/ml) demonstrated using **c** soft agar colony formation and **d** foci formation. Soft agar colony formation evaluated (upper) and images (lower) of colonies was captured after cultured in the complete medium over a period of 18 days. Images were captured under ×100 magnification using a bright field microscope. All assays were performed as described in Material and methods. The column is mean of triplicate experiments; bars, ±SD. **P < 0.001, *P < 0.05

To assess the potential reversibility of TFF3-driven oncogenic transformation of HMEC-*hTERT* cells, we generated stable clones of HMEC-*hTERT* cells with tetracycline-inducible *TFF3* expression and their control cells as described in Methods. Stable clones were designated as HMEC-*hTERT*-TetON-Dual2 and HMEC-*hTERT*-TetON-Dual2-TFF3 cells. Upon exposure to increasing concentrations of doxycycline (DOX), HMEC-*hTERT*-TetON-Dual2-TFF3 cells exhibited increased protein levels of TFF3, increased STAT3 activity and increased CCND1 and BCL2 protein levels (Fig. 7a). HMEC-*hTERT*-TetON-Dual2 cells did not exhibit changes in TFF3, STAT3 activity or CCND1 and BCL2 protein levels when exposed to increasing concentrations of DOX (SI 4A). Subsequently, HMEC-*hTERT*-TetON-Dual2-TFF3 cells also generated irregular, multi-acinar units with filled lumen when cultured in 3D Matrigel exposed to increasing concentrations of DOX. Moreover, HMEC-*hTERT*-TetON-Dual2-TFF3 cells exhibited an increased capacity for colonization in soft agar and foci formation compared to HMEC-*hTERT*-TetON-Dual2 cells when cultured in DOX (1 µg/ml) containing medium (Fig. 7c, d). Thus, DOX-induced TFF3 expression in HMEC-*hTERT* cells exhibited an oncogenic phenotype in Matrigel culture and an enhanced capacity for anchorage-independent growth.

**Fig. 8:**
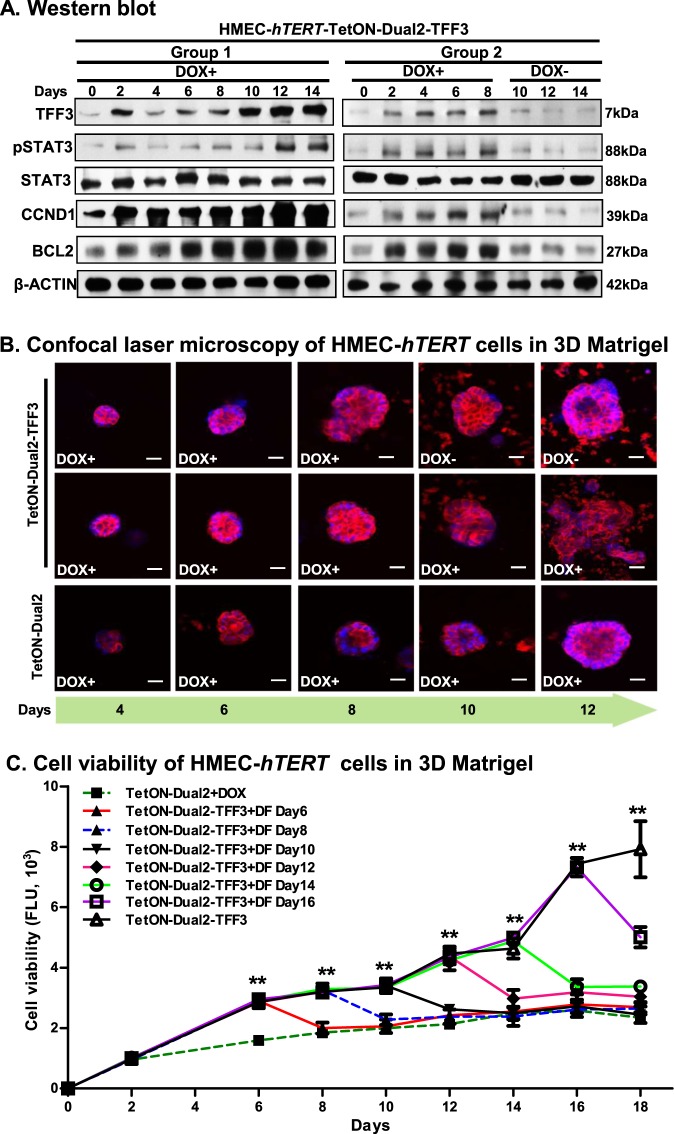
Depletion of inducible-TFF3 expression in HMEC-*hTERT* cells attenuates cell viability and efficiently normalized the acinar homoeostasis in 3D Matrigel HMEC-*hTERT*-TetON-Dual2-TFF3 cells were sub-divided into two sets; the first set was constantly cultured in the DOX-containing medium over a period of 14 days; whereas, the second set was cultured up to the eighth day in DOX-containing medium and thereafter cultured in DOX-free medium. **a** Western blot analysis was used to assess the protein levels of TFF3, STAT3 activity, CCND1, BCL2, and STAT3 in HMEC-*hTERT*-TetON-Dual2-TFF3 cells upon exposure to DOX (1 µg/ml). Soluble whole cellular extracts were run on an SDS-PAGE and immunoblotted as described in materials and methods. β-actin was used as an input control for cell lysate. The sizes of detected protein bands in kDa are shown on the right side. **b** Confocal laser scanning microscopic visualization of colonies generated by HMEC-*hTERT*-TetON-Dual2 and HMEC-*hTERT*-TetON-Dual2-TFF3 cells in 3D Matrigel with or without DOX in the medium. Confocal laser scanning microscopy was done using Rhodamine-conjugated phalloidin to visualize F-actin filaments (red colour), and nuclei were counterstained with DAPI (blue colour). **c** Cell viability of HMEC-*hTERT*-TetON-Dual2 and HMEC-*hTERT*-TetON-Dual2-TFF3 cells when cultured in 3D Matrigel with or without DOX in the medium. Sixth day onwards, every alternate day designated culture sets of HMEC-*hTERT*-TetON-Dual2-TFF3 was washed and then after cultured in the DOX-free medium. The cell viability of cells was evaluated using Alamar blue viability assay as described in Materials and methods. DF DOX-free. Chart point is mean of triplicate experiments; bars, ±SD. **P < 0.001, *P < 0.05

We next examined whether the effects of TFF3 on growth and acinar architecture of *immortalized*-HMEC-*hTERT* cells are reversible. Two groups of HMEC-*hTERT*-TetON-Dual2-TFF3 or HMEC-*hTERT*-TetON-Dual2 cells were cultured in DOX-containing complete medium. The first set of HMEC-*hTERT*-TetON-Dual2-TFF3 cells exhibited increased protein levels of TFF3 when cultured in the DOX-containing medium over the period of 14 days (Fig. 8a). Concomitantly, the protein levels of pSTAT3, CCND1, and BCL2 were also increased in HMEC-*hTERT*-TetON-Dual2-TFF3 cells when cultured in the DOX-containing medium. No change in the levels of total STAT3 protein was observed in HMEC-*hTERT*-TetON-Dual2-TFF3 cells when cultured in the DOX-containing medium over the same period. The second set of HMEC-*hTERT*-TetON-Dual2-TFF3 cells also exhibited increased protein levels of TFF3, pSTAT3, CCND1, and BCL2 over a period of 8 days when cultured in the DOX-containing medium. On the eighth day, this second set of HMEC-*hTERT*-TetON-Dual2-TFF3 cells were washed with prewarmed culture medium and afterward cultured in the DOX-free medium. The protein levels of TFF3 markedly declined in HMEC-*hTERT*-TetON-Dual2-TFF3 cells when reverted to DOX-free medium (Fig. 8a). Concomitantly, the levels of pSTAT3, CCND1, and BCL2 also decreased in HMEC-*hTERT*-TetON-Dual2-TFF3 cells when reverted to DOX-free medium. No changes were observed in STAT3 protein levels in HMEC-*hTERT*-TetON-Dual2-TFF3 cells when cultured in either DOX-containing or DOX-free medium (Fig. 7a). In contrast, HMEC-*hTERT*-TetON-Dual2 cells did not exhibit changes in the levels of TFF3 protein when cultured in DOX-containing medium over time (SI 4B).

In Matrigel culture, HMEC-*hTERT*-TetON-Dual2-TFF3 cells generated large, disorganized multi-acinar structures with filled lumina when cultured in the DOX-containing medium. Similar phenotypic effects on acinar architecture were observed with the second set of HMEC-*hTERT*-TetON-Dual2-TFF3 cells over the period of 8 days when cultured in the DOX-containing medium. However, when reverted to DOX-free medium, the acinar structures generated by HMEC-*hTERT*-TetON-Dual2-TFF3 cells increasingly regained a smoother appearance and loss of an aggressive cell phenotype as observed at 12 days (Fig. 8b). Moreover, in DOX-free medium, the overwhelming majority of acinar structures generated by second set of HMEC-*hTERT*-TetON-Dual2-TFF3 cells progressively resembled mature acini as generated by HMEC-*hTERT*-TetON-Dual2 cells, which eventually displayed prominent F-actin deposition at the periphery of spherical structures with a hollow lumen (Fig. 8b).

HMEC-*hTERT*-TetON-Dual2-TFF3 cells demonstrated cumulative increased cell viability over the period of 18 days when cultured in 3D Matrigel in DOX-containing medium compared to their vector control cells (Fig. 8c). On every alternate day after the sixth day, the HMEC-*hTERT*-TetON-Dual2-TFF3 cells were cultured in DOX-free medium. The viability of HMEC-*hTERT*-TetON-Dual2-TFF3 cells regressed and essentially reverted to basal levels of cell viability comparative to HMEC-*hTERT*-TetON-Dual2 cells cultured in DOX-containing medium (Fig. 8c). Thus, reduction of DOX-induced expression of TFF3 in HMEC-*hTERT* cells, previously grown with a DOX-induced expression of TFF3, resulted in efficient normalization of acinar architecture and concomitantly attenuated cell growth in Matrigel culture.

## Discussion

One paradigm of cancer development is that oncogenic cells are generated from normal cells by accumulation of genetic mutations that disrupt mechanisms limiting both cell proliferation and cell survival^22^. An alternative, hypothesis of cancer development proposes that deregulation of proliferation and enhanced cell survival creates “a platform that is both necessary and sufficient for the development of cancer” and genetic mutations are subsequently accumulated^2^. In support of this alternative hypothesis, we have previously described that autocrine growth hormone^20^, and herein we have demonstrated, that a single wild-type gene, TFF3, can transform *immortalized*-HMECs with tumour formation in vivo. This current study suggests that deregulation of two non-mutated wild-type genetic elements (*hTERT* and TFF3) is sufficient for the phenotypic conversion of normal human epithelial cells to cells with oncogenic characteristics. It should be noted that accumulation of further genetic mutations, a hallmark of cancer^22^, would presumably follow this initial transformation.

As an oestrogen-responsive gene, expression of TFF3 is also susceptible to environmental factors, such as endocrine disrupting chemicals, which are well-characterized to promote epigenetic modification, and are associated with long-term disease causation and progression, including neoplasia^7,8,31,32^. Hypomethylation-driven increases in TFF3 expression are positively associated with advanced clinicopathological features and progression of various human malignancies^33–36^. Hence a sustained and increased TFF3 expression associated with hypomethylation may promote aberrant cell proliferation and survival^37^. This is demonstrated herein by TFF3/STAT3-mediated upregulation of *BCL2* and *CCND1* in *immortalized*-HMECs. However, forced expression of BCL2 or CCND1 alone does not suffice for in vivo tumour formation^24,38^. TFF3 utilizes BCL2 protein as a critical survival mechanism^7^, and TFF3-stimulation apparently integrates the transcriptional response to promote both cell survival and proliferation in *immortalized* HMEC cells. This was also evident by the effect of combined inhibition of BCL2 and CCND1, which are both required for TFF3-driven transformation of *immortalized*-HMECs. Previously^39^ and herein, we observed that TFF3-driven STAT3 activity also downregulates *TP53*^40^. Continued cell cycle progression and decreased TP53-maintenance of genomic integrity would promote an accumulation of mutations^41–43^, which may further drive neoplastic transformation or progression^22,41^. Hence, accumulation of mutations may follow and be consequent to increased TFF3 expression in transformed cells with oncogenic phenotype.

We have demonstrated herein that termination of inducible-TFF3 expression in *immortalized*-HMECs reverses the TFF3-stimulated oncogenic phenotype. It remains to be determined whether the reversibility of TFF3-dependent oncogenic transformation in HMECs may only be observed in the short term or whether persistent and increased expression of TFF3 would result in accumulation of genetic mutations and partial or full irreversibility of the oncogenic phenotype upon TFF3 depletion or inhibition^44,45^. For example, it has been reported that despite dramatic regression of c-MYC inducible mouse mammary carcinoma after doxycycline withdrawal, there remains residual tumour cells that generate tumour recurrence independent of increased c-MYC expression^46,47^. Hence, future investigations are warranted to identify potential TFF3-mediated alterations in genomic stability. However, a plethora of evidence substantiates a potent role of TFF3 in cancer progression (see Introduction). Indeed, TFF3 acts as a promiscuous activator of multiple survival pathways in cancer cells, including HER1-4, PI3K/AKT, RAS/MEK/MAPK, and cSRC/STAT3, which are critically associated with enhanced cancer cell survival, metastasis, and resistance to therapy^8,11–18^. It is entirely plausible that a proportion of cancers that are intially dependent on TFF3 stimulation of oncogenic transformation for their establishment will later lose dependence on TFF3 for their progression. This is exemplified in histopathological studies of TFF3 expression where between 60^8^ and 83%^6^ of carcinoma are TFF3 positive. It is also possible that after establishment of a neoplastic growth, TFF3 assumes co-ordination of cellular functions involved in specific progression events such as metastasis or that TFF3 de novo assumes functional roles in survival of the metastatic deposits. Importantly, the propensity of cancer cells from TFF3-positive cancers to depend on TFF3-driven survival and dissemination represents a fundamental rationale for target-based therapeutic approaches against TFF3^48^. Indeed, TFF3 is highly expressed in the metastatic derivative colon carcinoma cell line (SW620) but not in the non-metastatic parental line (SW420)^49^ and locally invasive mammary carcinoma cells and mammary carcinoma cells in lymph nodes exhibit increased TFF3 expression compared to primary tumours^6^. Hence, TFF3 may possess distinct and dual roles in both oncogenic transformation and neoplastic progression.

In summary, we have demonstrated that the increased expression of TFF3 is sufficient to drive transformation of *immortalized*-HMECs to acquire an oncogenic phenotype.

## Materials and methods

### Cell culture and reagents

The human MC cell lines MCF10A and MCF12A were obtained from the American Type Culture Collection (ATCC, Rockville, MD) and HMEC-*hTERT* cells were obtained from Dr William C. Hahn (DF/HCC, USA). Cells were cultured as per ATCC propagation instructions. Human *TFF3*, *STAT3-siRNA*, and *STAT3 DN* constructs were previously described^7,8^. The luciferase reporter constructs for *α-2 macroglobulin*, *BCL2*, and the *CCND1* promoter was as previously described^8,20^. *siRNA*-oligo against *CCND1* or *BCL2* were purchased form LifeTechnologies (Singapore). STAT3 inhibitor, JSI-124, and Stattic, were purchased from Sigma-Aldrich (Singapore). Arcyriaflavin A (AA) and YC137 inhibitor were purchased from Santa-Cruz, USA. cSRC family kinase inhibitor PP1, and the specific cSRC kinase inhibitor PP2 was purchased from Sigma-Aldrich; the structurally related non-inhibitory PP3 (50 μM) was purchased from Calbiochem.

### Generation of stable clones with TFF3 expression

A pool of positive cell clones with stable forced expression of TFF3 in *immortalized*-HMECs was generated as previously described^8,20^. Briefly, positive transfectants were selected in 200–400 μg/ml G418 (Calbiochem) in the appropriate culture medium for the respective cell lines. Individual colonies were selected to determine TFF3 expression level by western blot analysis. Cell lines were established as HMEC-*hTERT*-TFF3, MCF10A-TFF3, and MCF12A, respectively, by pooling more than 15 individual colonies with high TFF3 expression. Tet-On® Advanced inducible gene expression system (Clontech Laboratories Inc, CA) obtained from Prof. Daniel G. Tenen at The Cancer Science Institute of Singapore (CSI), National University of Singapore (NUS), Singapore. For the inducible TFF3 expression system, forward and reverse oligonucleotides were annealed to produce the dsDNA, digested with BamHI and EcoRV and cloned into a *pTRE-Dual2* plasmid (Clontech Laboratories Inc, CA) and correct insertion and insert sequence checked by sequencing. Stable transfection of HMEC-*hTERT* cells with a plasmid containing *Tet-On advanced* and *pTRE-Dual2*-TFF3 was carried out using *X-tremeGENE HP DNA* transfection reagent, according to the manufacturer’s instructions (Clontech Laboratories Inc, CA). Stable pooled clones are designated as HMEC-*hTERT*-TetON-Dual2 and HMEC-*hTERT*-TetON-Dual2-TFF3 cells.

### PCR and quantitative-PCR

The patient cohort used herein consists of 53 specimens of invasive mammary ductal carcinoma that was previously described^8^. The histopathological diagnosis of the specimens was consistent with mammary carcinoma and was in accordance with World Health Organization guidelines. Isolation of total RNA, DNase I treatment, cDNA conversion, PCR, and qPCR was performed as previously described^8,35^. Gene analyses used herein and sequences of oligonucleotides are previously described^8,50^.

### Immunoblot and immunofluorescence

Immunoblot analysis was performed as previously described^8,35^, using rabbit anti-TFF3 polyclonal antibody. Mouse anti-β-actin, mouse anti-CDKN1A, mouse anti-BCL2, mouse anti-CDK2, rabbit anti-CDK4, rabbit anti-CCNE1, mouse anti-cSRC, and mouse anti-CCND1 antibody was obtained from Santa Cruz Biotechnology, CA. Rabbit anti-p-cSRC antibody were obtained from Cell Signaling, USA. Rabbit anti-pSTAT3(Tyr705) and mouse anti-STAT3 antibodies were obtained from Abcam, Cambridge, MA. Confocal microscopy scanning was performed as previously described^51^. Secondary antibody, Alexa Fluor 488 goat anti-rabbit IgG was purchased from Invitrogen, Singapore. Rhodamine-conjugated phalloidin (Sigma, St Louis, MO) was used to visualize f-actin filaments.

### Oncogenicity assays

Biological assays such as AlamarBlue® cell viability, BrdU incorporation, apoptotic activity, caspase 3/7 activity, soft agar colony formation, foci formation, lumen formation, and 2D & 3D (ex vivo) morphogenesis assay in Matrigel were performed as previously described^8,19,51^. Luciferase assays were performed as previously described^8,35,52^. Briefly, 5 × 10^5^ cells were transfected in a cell culture plate using *X-tremeGENE HP DNA* transfection reagent, according to the manufacturer’s instructions (Clontech Laboratories Inc, CA). Transfections were carried out in triplicate using 1 μg of the appropriate luciferase reporter construct and their control vector per transfection along with 0.1 μg of Renilla luciferase construct as a control for transfection efficiency. Luciferase activities were assessed using the Dual Luciferase Assay System (Promega Corp, Madison, WI, USA).

### Tumour xenograft

The xenograft assays followed the animal care protocol USTCACUC1301013, which was approved by The Institutional Animal Care and Ethics Committee of The University of Science and Technology of China. *Immortalized*-HMECs (MCF10A, MCF12A, and HMEC-*hTERT*) (4 × 10^6^ cells per site) with forced expression of TFF3 and their vector control cells were injected into the mammary fat pad of 4-week-old SCID-beige mice (Beijing Vital River Co, Beijing, China) (*n* = 8 for each) and followed procedures as previously described^20^. Histological analysis was carried out as previously described^8,20^.

### Statistics

All numerical data are expressed as mean ± SD from a representative experiment performed in triplicate. Statistical significance was assessed by using an unpaired two-tailed Student’s *t*-test or analysis of variance (*P* < 0.05 was considered as significant) by GraphPad Prism 5 (GraphPad Software, Inc, La Jolla, CA).

## Electronic supplementary material

SI1

SI2

SI3

SI4

Supplementary figure legends

## References

[1] Felsher DW (2003). Cancer revoked: oncogenes as therapeutic targets. Nat. Rev. Cancer.

[2] Green DR, Evan GI (2002). A matter of life and death. Cancer Cell.

[3] Elenbaas B (2001). Human breast cancer cells generated by oncogenic transformation of primary mammary epithelial cells. Genes Dev..

[4] Muskett FW, May FE, Westley BR, Feeney J (2003). Solution structure of the disulfide-linked dimer of human intestinal trefoil factor (TFF3): the intermolecular orientation and interactions are markedly different from those of other dimeric trefoil proteins. Biochemistry.

[5] Taupin D, Podolsky DK (2003). Trefoil factors: initiators of mucosal healing. Nat. Rev. Mol. Cell Biol..

[6] Ahmed AR, Griffiths AB, Tilby MT, Westley BR, May FE (2012). TFF3 is a normal breast epithelial protein and is associated with differentiated phenotype in early breast cancer but predisposes to invasion and metastasis in advanced disease. Am. J. Pathol..

[7] Kannan N (2010). Trefoil factor 3 is oncogenic and mediates anti-estrogen resistance in human mammary carcinoma. Neoplasia.

[8] Pandey V (2014). Trefoil factor 3 promotes metastatic seeding and predicts poor survival outcome of patients with mammary carcinoma. Breast Cancer Res..

[9] Lau WH (2015). Trefoil factor-3 (TFF3) stimulates de novo angiogenesis in mammary carcinoma both directly and indirectly via IL-8/CXCR2. PLoS ONE.

[10] Yamachika T (2002). Intestinal trefoil factor: a marker of poor prognosis in gastric carcinoma. Clin. Cancer Res..

[11] Dieckow J (2016). CXCR4 and CXCR7 mediate TFF3-induced cell migration independently from the ERK1/2 signaling pathway. Invest. Ophthalmol. Vis. Sci..

[12] May FE, Westley BR (2015). TFF3 is a valuable predictive biomarker of endocrine response in metastatic breast cancer. Endocr. Relat. Cancer.

[13] Chong Q. Y. et al. Release of HER2 repression of trefoil factor 3 (TFF3) expression mediates trastuzumab resistance in HER2+/ER+breast cancer. *Oncotarget* (2017) (In Press). 10.18632/oncotarget.18431 PMC5650333 29088778

[14] Taupin D (1999). The trefoil gene family are coordinately expressed immediate-early genes: EGF receptor- and MAP kinase-dependent interregulation. J. Clin. Invest..

[15] Kinoshita K, Taupin DR, Itoh H, Podolsky DK (2000). Distinct pathways of cell migration and antiapoptotic response to epithelial injury: structure-function analysis of human intestinal trefoil factor. Mol. Cell Biol..

[16] Taupin DR, Kinoshita K, Podolsky DK (2000). Intestinal trefoil factor confers colonic epithelial resistance to apoptosis. Proc. Natl Acad. Sci. USA.

[17] Rivat C (2005). Implication of STAT3 signaling in human colonic cancer cells during intestinal trefoil factor 3 (TFF3) -- and vascular endothelial growth factor-mediated cellular invasion and tumor growth. Cancer Res..

[18] Chen YH, Lu Y, De Plaen IG, Wang LY, Tan XD (2000). Transcription factor NF-kappaB signals antianoikic function of trefoil factor 3 on intestinal epithelial cells. Biochem. Biophys. Res. Commun..

[19] Debnath J, Muthuswamy SK, Brugge JS (2003). Morphogenesis and oncogenesis of MCF-10A mammary epithelial acini grown in three-dimensional basement membrane cultures. Methods.

[20] Zhu T (2005). Oncogenic transformation of human mammary epithelial cells by autocrine human growth hormone. Cancer Res..

[21] Schmeichel KL, Bissell MJ (2003). Modeling tissue-specific signaling and organ function in three dimensions. J. Cell Sci..

[22] Hanahan D, Weinberg RA (2011). Hallmarks of cancer: the next generation. Cell.

[23] Adiseshaiah P, Lindner DJ, Kalvakolanu DV, Reddy SP (2007). FRA-1 proto-oncogene induces lung epithelial cell invasion and anchorage-independent growth in vitro, but is insufficient to promote tumor growth in vivo. Cancer Res..

[24] Zhou Q (2000). Cyclin D1 overexpression in a model of human breast premalignancy: preferential stimulation of anchorage-independent but not anchorage-dependent growth is associated with increased cdk2 activity. Breast Cancer Res. Treat..

[25] Imbalzano KM, Tatarkova I, Imbalzano AN, Nickerson JA (2009). Increasingly transformed MCF-10A cells have a progressively tumor-like phenotype in three-dimensional basement membrane culture. Cancer Cell Int..

[26] Deshpande A, Sicinski P, Hinds PW (2005). Cyclins and cdks in development and cancer: a perspective. Oncogene.

[27] Yip KW, Reed JC (2008). Bcl-2 family proteins and cancer. Oncogene.

[28] Kamradt J (2003). Telomerase activity and telomerase subunit gene expression levels are not related in prostate cancer: a real-time quantification and in situ hybridization study. Lab. Investig..

[29] Silva CM (2004). Role of STATs as downstream signal transducers in Src family kinase-mediated tumorigenesis. Oncogene.

[30] Yu H, Pardoll D, Jove R (2009). STATs in cancer inflammation and immunity: a leading role for STAT3. Nat. Rev. Cancer.

[31] Ho SM (2012). Environmental epigenetics and its implication on disease risk and health outcomes. ILAR J..

[32] Zhang X, Ho SM (2011). Epigenetics meets endocrinology. J. Mol. Endocrinol..

[33] Okada H (2005). Frequent trefoil factor 3 (TFF3) overexpression and promoter hypomethylation in mouse and human hepatocellular carcinomas. Int. J. Oncol..

[34] Vestergaard EM (2010). Promoter hypomethylation and upregulation of trefoil factors in prostate cancer. Int. J. Cancer.

[35] Pandey V (2017). Hypomethylation associated enhanced transcription of trefoil factor-3 mediates tamoxifen-stimulated oncogenicity of ER+ endometrial carcinoma cells. Oncotarget.

[36] Busch M, Dunker N (2015). Trefoil factor family peptides--friends or foes?. Biomol. Concepts.

[37] Evan GI, Vousden KH (2001). Proliferation, cell cycle and apoptosis in cancer. Nature.

[38] Kelly PN, Strasser A (2011). The role of Bcl-2 and its pro-survival relatives in tumourigenesis and cancer therapy. Cell Death Differ..

[39] Niu G (2005). Role of Stat3 in regulating p53 expression and function. Mol. Cell Biol..

[40] Abbas T, Dutta A (2009). p21 in cancer: intricate networks and multiple activities. Nat. Rev. Cancer.

[41] Janssen A, Medema RH (2013). Genetic instability: tipping the balance. Oncogene.

[42] Loeb LA, Bielas JH, Beckman RA (2008). Cancers exhibit a mutator phenotype: clinical implications. Cancer Res..

[43] Lynch M (2008). The cellular, developmental and population-genetic determinants of mutation-rate evolution. Genetics.

[44] Weinstein IB, Joe AK (2006). Mechanisms of disease: oncogene addiction--a rationale for molecular targeting in cancer therapy. Nat. Clin. Pract. Oncol..

[45] Croce CM (2008). Oncogenes and cancer. N. Engl. J. Med..

[46] Felsher DW (2010). MYC inactivation elicits oncogene addiction through both tumor cell-intrinsic and host-dependent mechanisms. Genes & Cancer.

[47] Boxer RB, Jang JW, Sintasath L, Chodosh LA (2004). Lack of sustained regression of c-MYC-induced mammary adenocarcinomas following brief or prolonged MYC inactivation. Cancer Cell.

[48] Torti D, Trusolino L (2011). Oncogene addiction as a foundational rationale for targeted anti-cancer therapy: promises and perils. EMBO Mol. Med..

[49] Xue H (2010). Identification of serum biomarkers for colorectal cancer metastasis using a differential secretome approach. J. Proteome Res..

[50] Pandey V (2008). Autocrine human growth hormone stimulates oncogenicity of endometrial carcinoma cells. Endocrinology.

[51] Pandey V (2010). Artemin reduces sensitivity to doxorubicin and paclitaxel in endometrial carcinoma cells through specific regulation of CD24. Transl. Oncol..

[52] Pandey V (2010). Artemin stimulates oncogenicity and invasiveness of human endometrial carcinoma cells. Endocrinology.

