# Supplementary material for: Expression of two non-mutated genetic elements is sufficient to stimulate oncogenic transformation of human mammary epithelial cells
Source: Cell Death Dis. 2018 Nov 19;9(12):1147. doi: 10.1038/s41419-018-1177-6 (PMC6242831; doi:10.1038/s41419-018-1177-6)

Supplementary information 1

A. Soft agar colony formation

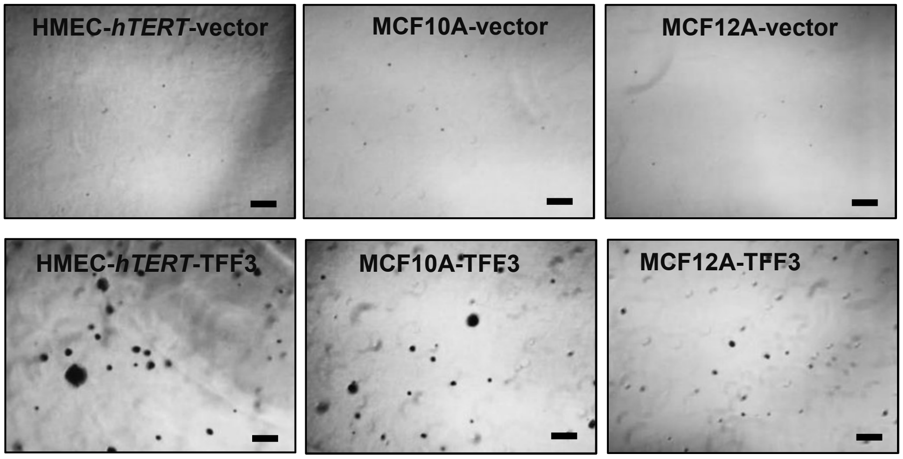

B. Suspension culture

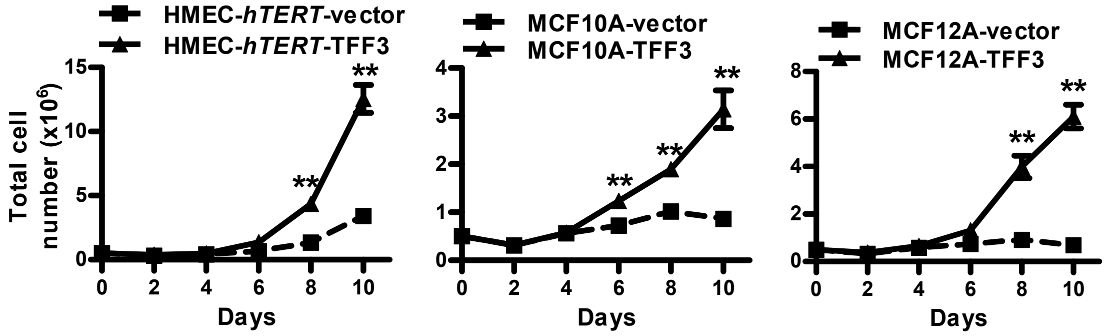

C. Cell viability

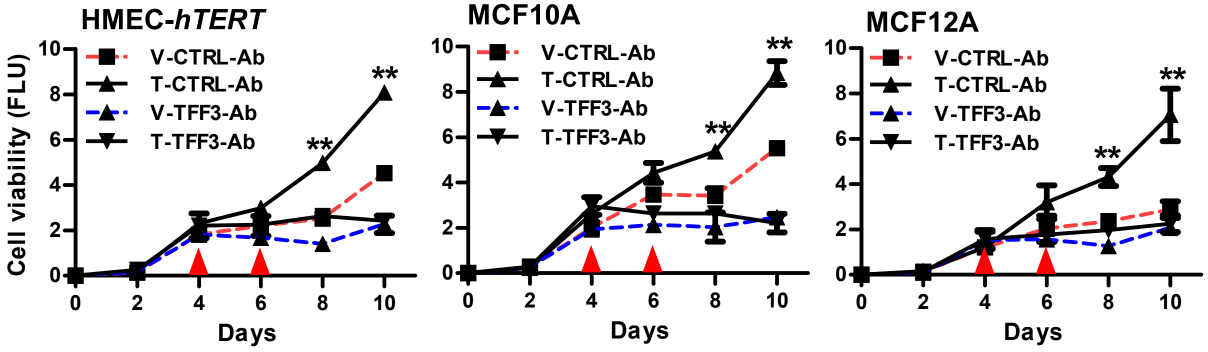

Supplement: Supplementary file 1 — SI1 [file 41419_2018_1177_MOESM1_ESM.pdf]
