# Supplementary figures and images for: Expression of two non-mutated genetic elements is sufficient to stimulate oncogenic transformation of human mammary epithelial cells
Source: Cell Death Dis. 2018 Nov 19;9(12):1147. doi: 10.1038/s41419-018-1177-6 (PMC6242831; doi:10.1038/s41419-018-1177-6)

## Supplementary information 2:

### A. Western blot

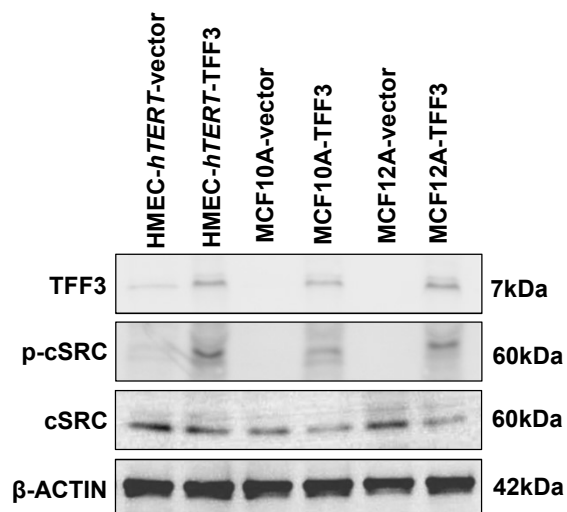

### B. Western blot

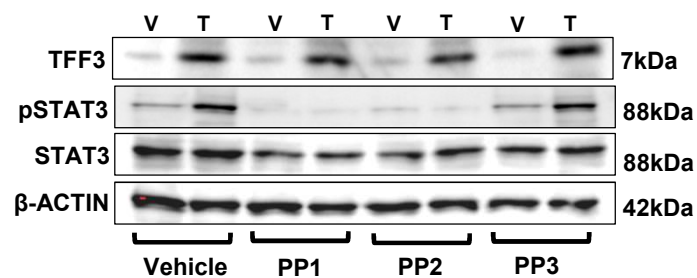

### C. Cell viability

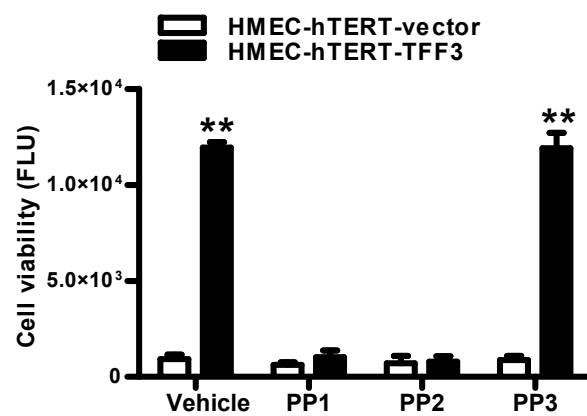

Supplement: Supplementary file 2 — SI2 [file 41419_2018_1177_MOESM2_ESM.pdf]

# Supplementary information 4:

## A. Western blot

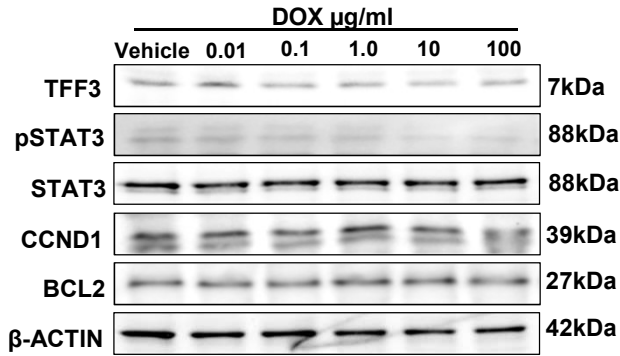

## B. Western blot

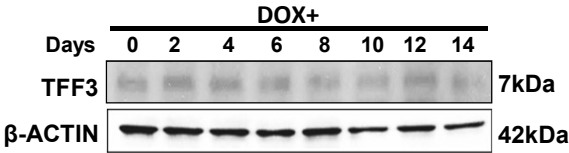

Supplement: Supplementary file 4 — SI4 [file 41419_2018_1177_MOESM4_ESM.pdf]
