# Supplementary material for: Expression of two non-mutated genetic elements is sufficient to stimulate oncogenic transformation of human mammary epithelial cells
Source: Cell Death Dis. 2018 Nov 19;9(12):1147. doi: 10.1038/s41419-018-1177-6 (PMC6242831; doi:10.1038/s41419-018-1177-6)
Supplement: Supplementary file 3 — SI3 [file 41419_2018_1177_MOESM3_ESM.pdf]

Supplementary information 3: qPCR analysis of the effect of forced expression of TFF3 in immortalized-HMECs (HMEC-*hTERT*, MCF10A, and MCF12A) on mRNA levels of several key genes associated with oncogenic transformation of human mammary epithelial cells.

		HMEC-*hTERT*		MCF10A		MCF12A	
**Functional Gene Grouping**	**Gene**	**Fold Change**	***p value***	**Fold Change**	***p value***	**Fold Change**	***p value***
**Cell Cycle Control & DNA Damage Repair**	***CCND1***	3.68	3.6E-04	17.35	8.0E-04	4.30	2.3E-03
	ATM	0.75	8.4E-04	4.80	2.2E-04	2.21	8.1E-04
	BRCA1	0.76	1.1E-02	5.95	4.0E-04	2.10	2.4E-04
	Cyclin E1	3.44	1.8E-05	8.89	9.1E-05	4.28	2.2E-04
	CDC25A	2.22	1.7E-04	22.16	4.1E-04	4.18	1.5E-04
	CDK2	1.16	3.0E-03	1.72	1.7E-02	2.79	1.1E-04
	CDK4	1.47	5.5E-03	2.47	5.8E-04	3.44	7.9E-05
	CDKN1A	0.34	5.6E-05	0.30	1.1E-03	0.32	6.5E-05
	CDKN2A	0.43	4.5E-02	0.57	4.2E-03	0.13	3.7E-04
	CHEK2	4.35	4.0E-03	5.71	1.7E-04	2.72	5.7E-04
	E2F1	1.73	4.4E-02	1.74	2.4E-02	2.82	1.3E-03
	MDM2	0.04	1.5E-02	10.63	2.6E-03	2.96	7.8E-03
	S100A4	4.27	4.1E-03	0.08	8.2E-03	0.09	6.0E-05
	TP53	0.09	1.2E-05	0.03	4.3E-06	0.03	2.6E-05
	CDKN1B	0.40	5.4E-04	0.53	3.0E-03	0.08	1.2E-05
**Apoptosis and Cell Senescence**	*APAF1*	1.45	1.3E-02	2.39	6.6E-03	0.05	6.0E-04
	BCLAF1	0.82	3.1E-02	2.39	5.1E-03	0.02	1.7E-04
	BAK1	1.37	9.1E-03	4.21	3.3E-04	0.02	2.0E-05
	BAD	12.26	5.3E-05	5.53	3.3E-03	69.50	4.7E-04
	BAX	0.47	2.5E-03	0.19	1.4E-03	0.24	5.9E-04
	BCL2	18.93	2.0E-03	56.83	1.8E-05	13.03	1.1E-03
	BCL2L1	3.45	1.9E-04	5.03	1.4E-04	3.57	6.3E-06
	CFLAR	4.59	2.1E-05	2.00	8.0E-03	0.21	7.9E-05
	CASP7	0.13	5.1E-05	0.09	8.8E-04	0.09	1.4E-05
	GZMA	2.06	1.5E-03	8.56	2.4E-05	0.32	4.5E-04
	HTATIP2	1.30	2.8E-02	1.49	1.4E-02	0.15	1.6E-03
	TERT	9.04	6.6E-04	20.76	5.0E-05	5.56	5.3E-04
	TNFRSF1A	2.77	5.5E-04	2.42	4.2E-02	0.27	1.7E-04
	TNFRSF25	1.27	1.3E-02	0.38	6.0E-03	0.12	1.3E-03
**Signal Transduction Molecules and Transcription Factors**	*AKT1*	1.38	5.8E-03	0.66	4.6E-03	1.17	5.9E-03
	ERBB2	0.71	5.8E-02	0.59	6.2E-03	3.26	4.5E-04
	ETS2	0.62	2.0E-03	2.00	7.4E-03	1.03	7.6E-02
	FOS	0.68	2.2E-02	0.18	2.9E-03	1.67	5.9E-03
	JUN	3.10	8.0E-04	10.85	3.5E-04	3.40	1.3E-03
	MYC	1.50	6.0E-03	3.22	1.5E-02	4.15	1.4E-03
	NFKB1	1.74	2.4E-03	1.82	2.8E-03	4.37	3.7E-03
	NFKBIA	1.55	4.8E-04	0.12	5.4E-04	2.33	6.5E-05
	PIK3R1	2.22	3.6E-04	0.23	1.0E-03	0.74	1.8E-02
	RAF1	1.71	2.2E-03	1.93	2.2E-02	1.55	1.3E-02
	SNCG	0.37	3.9E-03	0.30	7.0E-03	6.25	2.4E-04
	STAT3	1.05	2.6E-02	2.04	7.9E-05	2.25	4.7E-03
	STAT5B	0.55	2.5E-04	2.57	9.6E-04	1.34	6.9E-03
**Angiogenesis**	***ANGPT1***	2.09	1.5E-02	8.72	6.3E-03	11.16	4.2E-03
	ANGPT2	1.88	2.3E-03	3.07	2.1E-03	14.04	2.7E-03
	VEGF	3.58	2.0E-04	2.17	8.4E-03	1.99	1.6E-03
	COL18A1	2.51	8.6E-03	0.41	2.9E-03	0.74	2.2E-02
	IL8	20.21	8.2E-04	17.60	4.4E-05	46.51	4.3E-05
	TEK	1.53	2.3E-02	16.23	1.4E-04	6.24	2.2E-04
	TGFB1	2.35	9.7E-03	4.87	8.5E-04	1.24	4.7E-02
	TGBS1	0.03	2.0E-05	2.03	8.3E-04	0.33	2.7E-03
	TNF	0.84	2.9E-02	3.57	1.8E-03	0.01	1.5E-05
**Invasion and Metastasis**	***MET***	7.34	2.9E-05	6.92	6.7E-05	3.89	1.8E-04
	MMP1	0.05	8.9E-06	5.05	8.6E-05	12.66	5.1E-05
	MMP2	10.86	6.2E-04	3.15	4.3E-03	5.70	2.7E-03
	MTA2	0.37	4.2E-04	0.07	7.2E-04	0.64	4.5E-02
	NME1	1.31	3.3E-03	0.07	1.5E-03	12.83	4.0E-05
	PLAU	0.18	1.7E-04	17.91	2.3E-04	0.16	3.8E-03
	SERPINB5	0.05	5.6E-05	4.79	1.3E-02	4.17	8.8E-03
	SERPINE1	5.40	1.7E-04	4.37	8.2E-05	8.61	5.9E-04
	TIMP1	4.76	1.4E-05	6.56	4.9E-05	1.48	1.0E-02
	TIMP3	0.49	2.1E-04	3.83	3.5E-04	2.29	1.1E-04

Footnote: Change in gene expression is expressed as fold difference, respectively. Fold change values are representative of three independent biological experiments. To compensate for potential differences between markers, the relative expression was computed, based on the efficiency (E), normalized by a panel of housekeeping genes, *β-actin*, *HPRT*, and *GAPDH*. Gene indicated in bold were demonstrated similar directional changes at mRNA levels in all three HMECs with forced expression of TFF3 relative to their vector control cells.
