# Supplementary material for: Expression of two non-mutated genetic elements is sufficient to stimulate oncogenic transformation of human mammary epithelial cells
Source: Cell Death Dis. 2018 Nov 19;9(12):1147. doi: 10.1038/s41419-018-1177-6 (PMC6242831; doi:10.1038/s41419-018-1177-6)
Supplement: Supplementary file 5 — Supplementary figure legends [file 41419_2018_1177_MOESM5_ESM.pdf]

# Expression of two non-mutated genetic elements is sufficient to stimulate oncogenic transformation of human mammary epithelial cells

**Vijay Pandey[1,*], Min Zhang[2,*], Mingliang You[3], Weijie Zhang[2], Rumei Chen[3], Wei Zhang[4, 5], Lan Ma[1], Zheng-Sheng Wu[6], Tao Zhu[2], Xiu Qin Xu[7, #], and Peter E. Lobie[1, 3, #]**

[1]*Tsinghua-Berkeley Shenzhen Institute, Tsinghua University, Shenzhen, Guangdong, PR China;*

[2]*Hefei National Laboratory for Physical Sciences at Microscale and School of Life Sciences, University of Science and Technology of China, Hefei, Anhui, PR China;*

[3]*Cancer Science Institute of Singapore, National University of Singapore, Singapore;*

[4]*Department of Breast Surgery, The First Affiliated Hospital of Jinan University, Jinan University, Tianhe District, Guangzhou, Guangdong, PR China;*

[5]*Guangdong Provincial Key Laboratory of Malignant Tumor Epigenetics and Gene Regulation, Breast Tumor Center, Sun Yat-Sen Memorial Hospital, Sun Yat-Sen University, Guangzhou, PR China;*

[6]*Department of Pathology, Anhui Medical University, Hefei, Anhui, PR China;*

[7]*Institute of Stem Cell and Regenerative Medicine, Medical College, Xiamen University, Fujian, PR China;*

* Contributed equally

[#]**Correspondence to;** Xiu Qin Xu, PhD, Institute of Stem Cell and Regenerative Medicine, Medical College, Xiamen University, Fujian, PR China. E mail: xuxq@xmu.edu.cn. Peter E. Lobie, MD, PhD, Tsinghua-Berkeley Shenzhen Institute, Tsinghua University, Shenzhen, Guangdong, PR China. E-mail: pelobie@sz.tsinghua.edu.cn

**Running title**: TFF3 stimulates oncogenic transformation of *immortalized*-HMECs.

**Supplementary information 1:**

**(A)**. Representative bright field microscopy images of soft agar colonies were captured under X100 magnification.

**(B)** Total cell number in suspension culture by *immortalized*-HMECs with forced expression of TFF3 and their vector control cells. Cells were cultured in the complete medium over a period of 10 days.

**(C)** Determination of cell viability of *immortalized*-HMECs with forced expression of TFF3 and their vector control cells in three-dimensional Matrigel culture, on treatment with rabbit anti-TFF3 polyclonal antibody (pAb) (500μg/ml) or control. Red colour arrowheads indicate the day of pAb addition to the medium.

All assays were performed as described in Material and Methods. Chart point is mean of triplicate experiments; bars, ±SD. $^{**}P < 0.001$, $^{*}P < 0.05$.

**Supplementary information 2: Forced expression of TFF3 in *immortalized*-HMEC cells enhanced phosphorylation of cSRC that subsequently increased STAT3 activity to promote cell viability.**

**(A)** Western blot analysis was used to assess the levels of TFF3, p-cSRC (Tyr416) and cSRC in *immortalized*-HMEC (HMEC-*hTERT*, MCF10A, and MCF12A) cells with forced expression of TFF3. **(B)** Western blot analysis was used to assess the levels of TFF3, pSTAT3, and STAT3 in HMEC-*hTERT* cells with forced expression of TFF3 on exposure to PP1 (5μM), PP2 (2μM) or PP3 (50μM). **(C)** Cell viability of HMEC-*hTERT*-vector and HMEC-*hTERT*-TFF3 cells on exposure to PP1 (5μM), PP2 (2μM) or PP3 (50μM).

Soluble whole cellular extracts were run on an SDS-PAGE and immuno-blotted as described in materials and methods. β-ACTIN was used as an input control for cell lysate. The sizes of detected protein bands in kDa are shown on the right *side.* All assays were performed as described in Material and Methods. The column is mean of triplicate experiments; bars, ±SD. $^{**}P < 0.001$, $^{*}P < 0.05$.

**Supplementary information 3:**

qPCR analysis of the effect of forced expression of TFF3 in immortalized-HMECs (HMEC-*hTERT*, MCF10A, and MCF12A) on *mRNA* levels of several key genes associated with oncogenic transformation of human mammary epithelial cells.

**Supporting information 4:**

**(A)** Western blot analysis was performed to assess the levels of TFF3, pSTAT3, CCND1, BCL2, and STAT3 in HMEC-*hTERT*-TetON-Dual2 cells after exposure to increasing concentrations of DOX (0.01 to 100ug/ml). **(B)** Western blot analysis was performed to assess the levels of TFF3 in HMEC-*hTERT*-TetON-Dual2 cells when cultured in DOX-containing (1μg/ml) medium over time.

Soluble whole cellular extracts were run on an SDS-PAGE and immuno-blotted as described in materials and methods. β-ACTIN was used as an input control for cell lysate. The sizes of detected protein bands in kDa are shown on the right *side.*
